# Metrics and evaluation of scientific productivity: would it be useful to normalize the data taking in consideration the investments?

**DOI:** 10.1186/s12934-019-1236-4

**Published:** 2019-10-26

**Authors:** Ario de Marco

**Affiliations:** Laboratory of Environmental and Life Sciences, University of Nova Gorica, Vipavska cesta 13, Rožna Dolina, 5000 Nova Gorica, Slovenia

**Keywords:** Research metrics, Research economy, Research fund distribution

## Abstract

There has been in increasing interest in evaluating research production by means of “objective parameters” which should score the scientific impact of single articles and researchers’ career. In contrast, the attention of the economic aspects of research production has been highly neglected. I suggest that introducing the assessment of the return of research investment would be useful for fair comparison among researchers and probably it would render more understandable to public opinion what are the criteria according to which research funds are distributed.

## Background

**Fig. 1:**
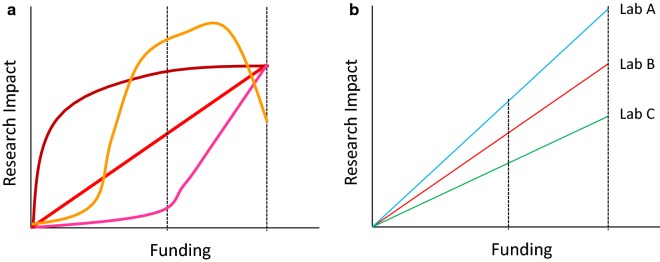
Hypothetical profitability curves for research investments. The successive funding amounts allocated to a specific lab could result into a variable scientific impact (**a**) and such relationship could vary among labs (**b**). The reported curves are hypothetical because no (published) investigation has been performed so far which could illustrate the relationship between investments and r return in terms of research impact

The debate concerning which parameters should be considered in order to objectively evaluate the impact of research work is pertinent because the resulting scores are critical to obtain funding and for career advancement. The attention of metrics specialists has mainly focused on the search for values that better reflect the “quality” and the impact of published papers, such as the number of citations and downloads, composite indexes of different complexity, quotations in different media and sometimes even social indicators [1–3]. In contrast, I have never come across a consideration of the production costs of the scientific work: how much grant money has been invested to obtain those data? How many euros were necessary to obtain a single citation? Of course, the absolute value of the published papers will not change, but the evaluation of researchers could be modified according to her/his capacity to produce fruitful research with a determined budget. Here I do not wish simply recall the important aspects related to omics data usability [4], namely the necessity to record the experimental results in formats which enable their metadata analysis and maximize the initial investment to collect them. I’d rather ask the question whether the curve relating funding amounts and “research impact” is linear or not (Fig. 1) to understand what is the best way to distribute the resources among the stakeholders and if, in the same “income class”, there are highly different level of investment profitability.

## Main text

At present, the weight of accumulated attributes strongly affects the opportunity to win further funds, despite the declarations of many agencies to consider only the intrinsic value of the submitted proposals and not the “treasure” represented by the previous achievements of the proposer. In the practice, very often the past guaranties for the future. This approach has its (conservative) logic because should minimize the risk for the funding agency but is it possible that it rewards the inertia of what has already been acquired rather than clarifying whether the researcher is still as active as she/he was in the past? Furthermore, there is another point: are h-index, publications on high IF journals or number of citations per se reliable methods to assess researchers’ past merits? It is clear to anybody that having more funding enables hiring more collaborators, buying better equipment, accessing to more effective services and this will result in better and more publications. So, once won the first pot, the person will have better chances to produce interesting results which in return will allow the access to further funds. As any self-feeding system, it does not give guarantees of efficiency. Furthermore, are the “objective parameters” used to calculate the accomplishments meaningful tools to evaluate the more recent performance? Research requires the preparation of collections and reagents—and this is typically the case of microbiology and protein biotechnology fields- which should be funded because represent a long-term investment for the whole community but that in contrast are activities often disregarded and not rewarded. I anticipate that a normalized evaluation of research and of researchers (i.e., “fair” metrics you prefer divided by the available funding obtained in that period) would be useful to assess the capacity of a researcher to exploit her/his resources, a quality that should be considered. This parameter would not modify the quality of a proposal but integrate the overall evaluation of researchers, indicating how active and efficient they have been in a specific period of time and how effective they were in exploiting their budgets. Such value could be even multiplied by a factor which considers the indirect contribution of the infrastructures and supporting facilities at the host institution. During the selection process for the assignment of research funds, this parameter would serve to foresee how productive someone would be if that money would be granted. In this proposal there is no punitive desire, but the curiosity to know if there is a “funding limit” that once reached decrees that further investments in that lab will be wasted rather than synergistically exploited (Fig. 1). Probably, funding agencies should share my curiosity.

## Conclusions

I have no data to predict how the implementation of such proposal could impact the fund distribution but expect that it could contribute to a more equal repartition between rich and marginal research centers. Research advancement needs visionary projects that can be realized only inside perfectly equipped institutes and can be very expensive because are high-risk for definition. But it also profits from methodological optimization and repeats and needs the widespread training of students even in peripheral research labs. Both top and routine research activity can be performed optimally or poorly and I find that it would be fair rewarding the achievements also considering the means with which they were obtained, not only according to their absolute impact.

## Data Availability

Not applicable.
